# A Comparative Study of Palatal Rugae Patterns among Igbo and Ikwerre Ethnic Groups of Nigeria: A University of Port Harcourt Study

**DOI:** 10.1155/2014/123925

**Published:** 2014-09-08

**Authors:** P. C. Ibeachu, B. C. Didia, A. O. Arigbede

**Affiliations:** ^1^Department of Human Anatomy, College of Health Sciences, University of Port Harcourt, PMB 5323, Choba, Rivers, Nigeria; ^2^Department of Restorative Dentistry, College of Health Sciences, University of Port Harcourt, Choba, Rivers State, Nigeria

## Abstract

*Background.* Palatal rugae pattern of an individual is species specific and is said to be as unique as finger print. *Aims and Objectives.* The aim of this study was to identify and compare the rugae pattern of Igbo and Ikwerre populations in Nigeria for human identification purposes. *Materials and Methods.* The present study was conducted from random sampling of University of Port Harcourt students. A total number of 140 subjects (70 Igbos and 70 Ikwerres) of age bracket of 18–30 were used. *Results.* The different shapes of rugae were obviously observed with varying degrees of predominance among the two tribes. The most predominant patterns are wavy and curvy followed by straight. The Igbos were predominantly wavy while the Ikwerres were predominantly of curve and straight patterns. The Igbo males and females had more wavy pattern with percentage values of 51.6% and 59.9% which is significantly higher in proportion (*P* < 0.05) as compared to the Ikwerre males and females with percentage values of 35.6% and 40.6%. On the other hand, Ikwerre males and females proved to be curve dominant with percentage values of 45.2% and 34.4% and this showed significant difference in proportion (*P* < 0.05) as compared to the Igbo males and females with percentage curve values of 27.9% and 26.1%. *Conclusion.* The result obtained from this study is clear evidence of ethnic differences in relation to sex; hence the incidence of predominance is population dependent.

## 1. Introduction

The appropriate method for human identification is of paramount importance in the field of forensics science. Palatal rugae are epithelial ridges on anterior part of palatal mucosa on each side of mid-palatine raphe behind incisive papilla. They are protected from trauma and high temperatures because of their internal position in the oral cavity, surrounded and protected by lips, cheeks, tongue, teeth, and bone (Kamala et al. [1]). In the literature there is consensus opinion that palatal rugae remain fairly stable in number and do not undergo any change due to growth, ageing, tooth extraction, and disease [2–4]. Morphological changes may occur due to trauma, surgery, persistent pressure, and proliferative benign and malignant lesions [4]. Once formed, it only changes in its length, due to normal growth, staying in the same position throughout the life of a person (Bharath et al. [5]). combination patterns amongst the Palatal rugae have been studied for various reasons, most important one being personal identification [4]. Though rugoscopy can be used in forensic identification, few studies have questioned this application in patients undergoing orthodontic treatment and in edentulous patients because of the chances of change in rugae pattern over a period of time in these patients [6, 7]. Nevertheless in patients undergoing orthodontic treatment, changes in the length of the rugae have been reported while the pattern remains unchanged [2, 6]. Palatal rugae appear to possess the features of an ideal forensic identification parameter because of its uniqueness, postmortem resistance, and stability. In addition, rugae pattern may be specific to racial and sex groups facilitating population identification which may be required after a disaster. In fact, differences in rugae pattern have been found in relatively similar population groups (Kapali et al. [8]). It has been reported that no similarity exists in palatal rugae patterns of siblings, twins, and even their parents (Faisal et al. [9] and Kamala et al. [1]). Studies have shown that certain rugae patterns are specific to a particular population (Kapali et al. [8], Nayak et al. [10], Arora et al. [11], Bajracharya et al. [4], Kiran Shetty et al. [12], and Shetty et al. [13]).

Hence palatal rugae might be of immense help in identification of individuals in Forensic Odontology, provided both ante- and postmortem details are available Bajracharya et al., [4].

However, the use of palatal rugae patterns in human identification has not been reported in the population of study. This work is therefore aimed at investigating the different rugae patterns among Igbo and Ikwerre ethnic groups of Nigeria.

## 2. Materials and Methods

The study consisted of 140 healthy human subjects between 18 and 35 years of age who were randomly selected. The sample size is comprised of 70 Igbo (males and females) 70 Ikwerre (males and females). The subjects were briefed regarding the procedure and nature and only those who gave their informed consent and showed keen cooperation participated in the study. Questionnaire method was used to ascertain their ethnicity (three generations proved ethnicity), family history, and health issues. This study was conducted at the dental center of the University of Port Harcourt Teaching Hospital, Rivers State, South-South Nigeria.

### 2.1. Ethical Considerations

Research Ethics Committee of the College of Health Sciences, University of Port Harcourt, approved the study.

### 2.2. Exclusive Criteria

The subjects with congenital anomalies/malformations, previous orthognathic surgery, bony and soft tissue protuberances, active lesions, deformity or scars, and trauma of the palate were not selected. Also, subjects who were wearing partial dentures and braces were excluded.

### 2.3. Inclusive Criteria

Normal subjects, whose parents and grandparents are of Igbo and Ikwerre origins within University of Port Harcourt, are included.

### 2.4. Methodology

The subjects were made to sit upright on the dental chair. The alginate paste was prepared by mixing the alginate impression powder (Spofadental, A Kerr Company, LOT: 2302461) with water as instructed by the manufacturer. Maxillary impressions of the subjects were taken using a perforated impression tray loaded with an alginate. Dental casts were made with type 4 dental stone (Spofadental A Kerr Company, LOT: 4511215). The rugae were highlighted by a black pen marker on the cast under spotlight and recorded while the length was measured with a digital caliper calibrated to 0.0 mm.

### 2.5. Method of Identification

The study was based on the classification given by Thomas and Kotze [14] and Kapali et al. [8].

*Length of Rugae.* The length of rugae is as follows:

 fragmentary (<3 mm),
 secondary (3–5 mm),
 primary (>5 mm).

*Shape of Rugae.* The shapes of individual rugae were classified into 4 major types.

 Curvy: the curved type had a simple crescent shape with a gentle curve.
 Wavy: the wavy rugae were serpentine (snake-like) in shape.
 Straight: the straight types ran directly from their origin to insertion.
 Circular: they are classified as rugae that showed definite continuous ring formation.

*Unification.* This occurs when two rugae are joined at their origin or termination. Unification is classified into two categories.

 Diverging: rugae were considered to be diverging if two rugae had the same origin but immediately branched.
 Converging: rugae were considered to be converging if two rugae with different origins join on their lateral portions.

### 2.6. Statistical Analysis

The total numbers of the various rugae patterns were counted, the percentages were recorded, and pie charts were used to describe the distribution of the various patterns within the tribes. *Z*-test was used to compare the difference in the mean values of the classified length as well as proportionality differences in distribution of the patterns in the ethnic groups. The associations between the distributions of the different rugae shapes with ethnicity in relation to sex were tested using chi-square analysis. *P* value ≤0.05 is considered as being statistically significant.

## 3. Results

**Figure 1:**
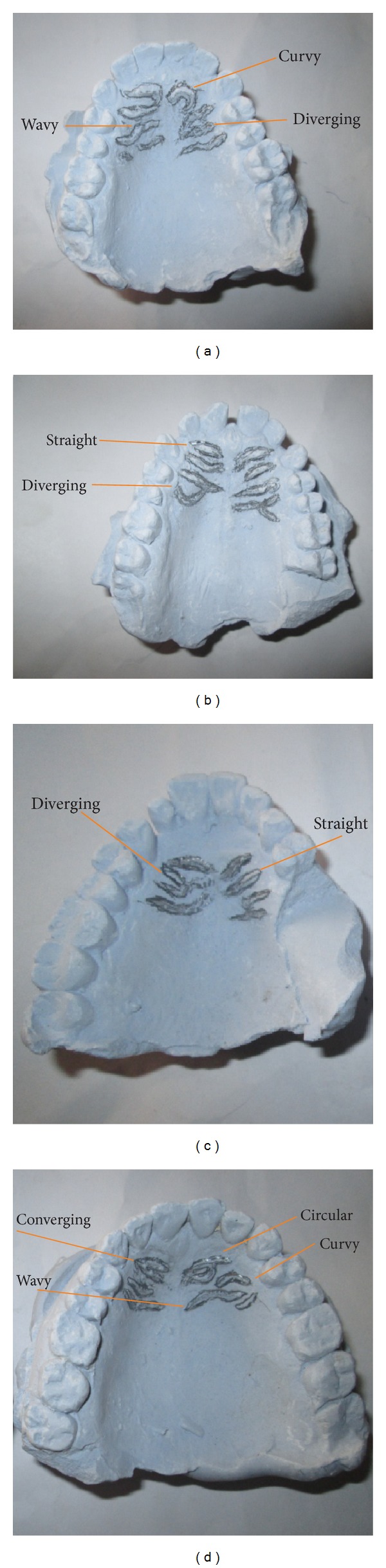
Maxillary cast showing the rugae patterns.

**Figure 2:**
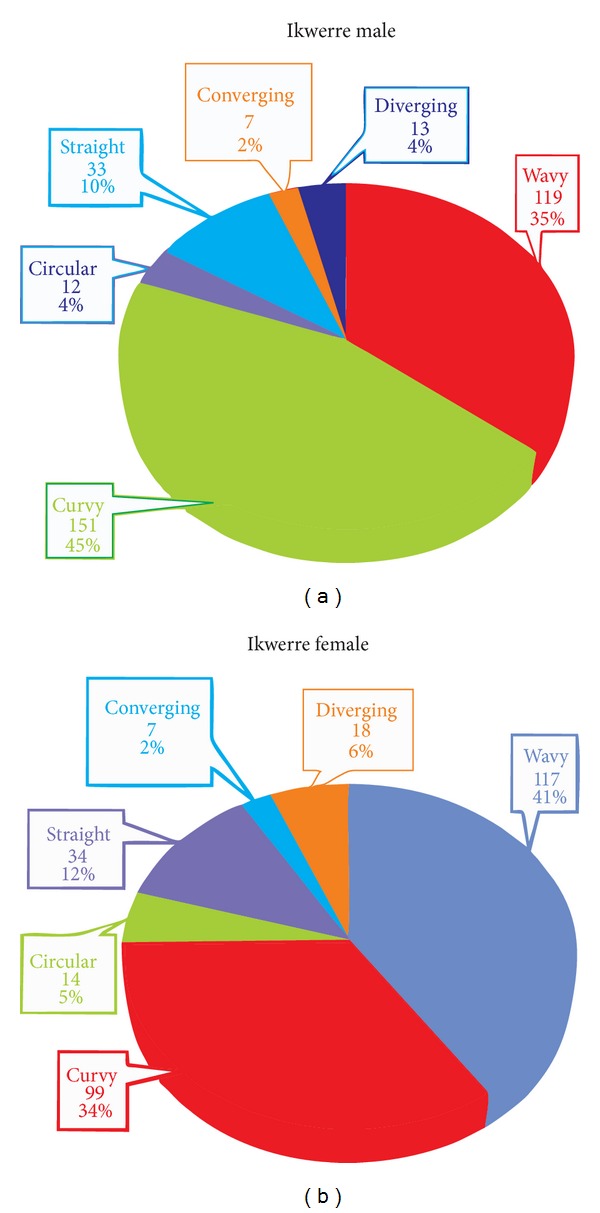
(a) Percentage distribution for Ikwerre males, (b) percentage distribution for Ikwerre females.

**Figure 3:**
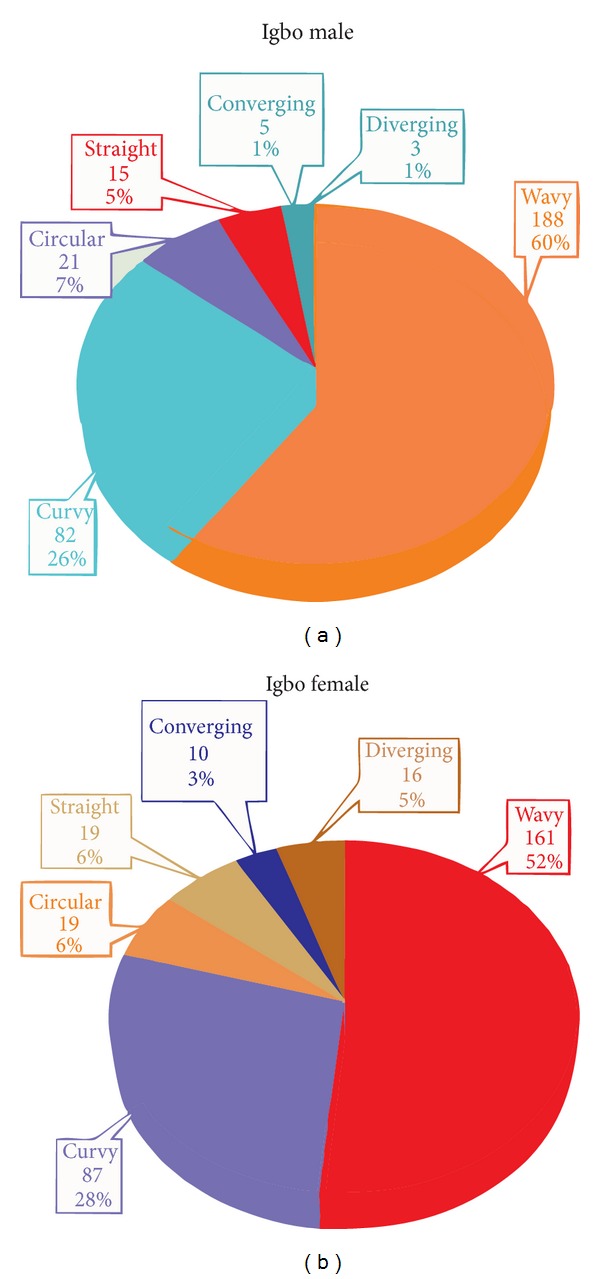
(a) Percentage distribution for Igbo males, (b) percentage distribution for Igbo females.

The rugae patterns were uniquely structured and patterned in all the individuals, and there was no evidence of similarity in the combination patterns amongst the tribes (Figure 1). The total number of various rugae patterns and their percentages are shown in Figures 2 and 3. The Igbo females have more rugae than the Ikwerre females while the Ikwerre males have more rugae than the Igbo males. The different shapes of rugae were obviously observed with varying degrees of predominance. In decreasing order of predominance are the wavy, curvy, and straight patterns. The circular pattern and unification of rugae were less common in both tribes. We observed some degree of ethnic variability in the proportionality of some rugae shapes which was statistically significant (*P* > 0.05). The wavy pattern in Igbo males and females was significantly higher in proportion than the Ikwerre males and females at *P* < 0.05, while the Ikwerre males and females have more curvy patterns which is significantly higher in proportion than the Igbo males at *P* < 0.05. The Ikwerre female showed high proportion than the Igbo female for the straight and unification (diverging) rugae shape which was statistically significant (*P* < 0.05) (Tables 1 and 2).

The distribution of the total number of individual rugae pattern in Igbo and Ikwerre did not show any significant association except in the unification (diverging) rugae (Table 3). There was significant association in distribution of total number of rugae pattern in Igbo and Ikwerre in relation to sex (Table 4). Chi-square analysis of individual rugae patterns based on sides and total sides did not show any significant association (Tables 5 and 6). The analysis of rugae length showed that primary rugae were more in Ikwerre while secondary rugae were more in Igbo although primary rugae dominated. The male Ikwerre had significantly higher mean value of primary rugae than the male Igbo (*P* = 0.011) while female Ikwerre had an insignificantly higher number of primary rugae than female Igbo (Table 7).

## 4. Discussion

The uniqueness and stability of palatal rugae as an adjunct of human identification have been well known and implemented in the field of forensics and orthodontics [8, 15]. Despite the controversy about the stability of qualitative and quantitative characteristics of rugae and the extent of differences between ethnic groups and sex, the uniqueness to individuals has been recognized in forensic science as providing potential source of identification [8].

The present study evaluated the different shapes of rugae, level of predominance, combination pattern, total number of rugae, and length of rugae. The various rugae shapes were duly represented with varying degrees of predominance. The most predominant rugae pattern was the wavy followed by curvy and straight, while circular and unification of rugae were obviously less common in both populations. There was no similarity in the combination patterns which confirms its individualistic nature. The high incidence of predominance in wavy and curvy patterns has been reported by Nayak et al. [10], Kotrashetti et al. [16], Kumar et al. [17], Surekha et al. [18], Shanmugam et al. [19], Mohammed et al. [20], Bajracharya et al., [4], and Kapali et al. [8], and this effect could be regarded as dominant pattern in most populations. The study, however, found considerably higher number of straight patterns in Ikwerre which is in accordance with Shanmugam et al. [19] and Paliwal et al. [21] but in contrast to the finding by Kallianpur et al., [6] who reported the straight type to be the second predominant pattern in Nepalese population, and Rath and Reginald [22] in their study of palatal rugae. Generally, the Igbo populations are predominantly wavy in pattern while the Ikwerre populations are predominantly curvy and straight in pattern. The rugae pattern in males and females of these populations followed a specific trend. Putting the predominant patterns into consideration, the Igbo males and females had more numbers of wavy patterns which is significantly higher in proportion when compared with Ikwerre males and females. This, however, agrees with Kiran Shetty et al., [12] who said that the comparison for two populations revealed that there was a significant difference between Malayalees and Kodavas for wavy and unification patterns. The Ikwerre males and females had more curvy patterns which is also statistically significant when compared with Igbo males and females, which agrees with Surekha et al., [18] who found that the curved pattern was predominant in Manipuri population than in Kerala and was statistically significant. These findings also agree with Shetty et al., [13] who revealed that Indian males had more curvy patterns than Tibetan males while Tibetan females had more numbers of wavy patterns than Indian females. The Igbo males had more circular patterns which is statistically insignificant than Ikwerre males while the Ikwerre females had more straight patterns with high significant difference in proportion than the Igbo females. These significant differences in proportionality found in wavy, curvy, and straight patterns could be attributed to population differences due to environmental factors. Although there were ethnic peculiarities with respect to significant level of proportionality in shapes, chi-square test, however, did not show significant association in the pattern of distribution of the various rugae shapes among the tribes. The distribution patterns were randomly influenced. The total number of rugae in male and female Igbo population showed no significant difference which is in accordance with Bharath et al. [5] in the Indian study and studies by Faisal et al. [9] in the Saudi population and Shetty and Premalatha [23] in the Mangalorean population but in contrast with Dohke and Osato [24] in their studies in Japanese population and Shetty et al. [13] in Mysorean and Tibetan populations which revealed that females had fewer number of rugae than males. The Ikwerre population showed that males had more rugae than the females, though insignificant but agrees with Dohke and Osato [24]. The right and left distribution of rugae pattern revealed that males and females of the two populations had more rugae on the left as compared to the right and this contradicts with Kapali et al. [8], Paliwal et al. [21], and Madhankumar et al. [25] who did not observe much difference between the left and right sides in their various population. The higher number of rugae on left side agrees with study done by Surekha et al. [18], Kallianpur et al. [6], S. Goyal and S. Goyal [26], and Bajracharya et al. [4] who explained that it was due to phenomenon of regressive evolution dominating the right side of the palate and being more evident in females. Also, the Igbo females had more rugae on both right and left than Ikwerre females while the Ikwerre males had more rugae on both right and left than Igbo males. The Igbo males had more unification patterns of rugae (converging and diverging) than the Ikwerre males while the Ikwerre females had more unification patterns of rugae than the Igbo females, with significant difference in proportion in diverging pattern. These findings are in disagreement with Manjunath et al., [27] who said that comparisons of the unification of rugae both converging and diverging did not show any specific trend. The evaluation of rugae length showed that they were basically primary rugae. The males in both populations had more primary rugae than their females and this observation corresponds with that made by Shetty et al. [13] who reported that the Mysorean males had more numbers of primary rugae than their female counterparts. The analysis of rugae length showed that primary rugae were more in Ikwerre while secondary rugae were more in Igbo although primary rugae dominated. This finding is in accordance with Surekha et al. [18] who said that primary rugae were considerably longer in Kerala population than in Manipuri population, whereas secondary rugae were longer in Manipuri population. The palatal rugae shape and length showed clear evidence of discriminant characteristics in these two tribes. When genetically similar populations were considered for differentiation, continuous variables such as rugae measurement may have its limitations; therefore discrete variables such as rugae shapes could provide better result (Nayak et al. [10], Shanmugam et al. [19], and Rath and Reginald [22]).

## 5. Conclusion

The individualistic nature of palatal rugae patterns and its ethnic variability were evident in this study. The patterns of Igbo were predominantly wavy while those of Ikwerre were curvy and straight. This shows that every population including Igbo and Ikwerre has unique predominant pattern with individual differences. The rugae pattern could therefore be a strong forensic tool in analyzing races, provided antemortem records are available. Further studies from other ethnic groups in Nigeria are therefore required to validate these findings.

## Figures and Tables

**Table 1:** *Z*-test of proportionality difference in rugae pattern distribution between Ikwerre and Igbo males.

Pattern	Tribe	Obs. population (*n*)	Sampled population (*N*)	Obs. proportion	*z*-value (calculated)	|*z*| critical	*P* value (obs.)	Inference
Wavy	Ikwerre	119	335	0.356	−3.98	1.96	<0.0001	**Significant difference in proportions** ∗
	Igbo	161	312	0.511				

Curvy	Ikwerre	151	335	0.451	4.53	1.96	<0.0001	**Significant difference in proportions** ∗
	Igbo	87	312	0.279				

Circular	Ikwerre	12	335	0.036	−1.49	1.96	0.136	No significant difference in proportions
	Igbo	19	312	0.061				

Straight	Ikwerre	33	335	0.099	1.76	1.96	0.078	No significant difference in proportions
	Igbo	19	312	0.061				

Converging	Ikwerre	7	335	0.021	−0.89	1.96	0.373	No significant difference in proportions
	Igbo	10	312	0.032				

Diverging	Ikwerre	13	335	0.039	−0.77	1.96	0.441	No significant difference in proportions
	Igbo	16	312	0.051				

The asterisks were used to demonstrate values with significant difference.

**Table 2:** *Z*-test of proportionality difference in rugae pattern distribution between Ikwerre and Igbo females.

Pattern	Tribe	Obs. population (*n*)	Sampled population (*N*)	Obs. proportion	*z*-value (calculated)	|*z*| critical	*P* value (obs.)	Inference
Wavy	Ikwerre	117	289	0.405	−4.7572	1.96	<0.0001	**Significant difference in proportions** ∗
	Igbo	188	314	0.599				

Curvy	Ikwerre	99	289	0.343	2.1791	1.96	0.02926	**Significant difference in proportions** ∗
	Igbo	82	314	0.261				

Circular	Ikwerre	14	289	0.048	−0.9672	1.96	0.33204	No significant difference in proportions
	Igbo	21	314	0.067				

Straight	Ikwerre	34	289	0.118	3.1373	1.96	0.00168	**Significant difference in proportions** ∗
	Igbo	15	314	0.048				

Converging	Ikwerre	7	289	0.024	0.7289	1.96	0.4654	No significant difference in proportions
	Igbo	5	314	0.016				

Diverging	Ikwerre	18	289	0.062	3.5282	1.96	0.00042	**Significant difference in proportions** ∗
	Igbo	3	314	0.01				

The asterisks were used to demonstrate values with significant difference.

**Table 3:** Association of tribe and the distribution of individual rugae shape with sex.

	Tribe		DF	Chi square (*χ* ^2^)	Critical value	Chi (*ρ*) calculated *P* value	Inference
	Igbo	Ikwerre					
Wavy							
Male	161	119	1	1.039	3.84	0.31	No significant association
Female	188	117					
Curvy							
Male	19	12	1	0.011	3.84	0.91	No significant association
Female	21	14					
Circular							
Male	87	151	1	3.270	3.84	0.07	No significant association
Female	82	99					
Straight							
Male	19	33	1	0.397	3.84	0.53	No significant association
Female	15	34					
Converging							
Male	10	7	1	0.829	3.84	0.36	No significant association
Female	5	7					
Diverging							
Male	16	13	1	8.642	3.84	0.003	**Significant association** ∗
Female	3	18					

The asterisks were used to demonstrate values with significant difference.

**Table 4:** Association between the tribe and sex with rugae pattern distribution.

	Ikwerre		Igbo		DF	Chi-square (*χ* ^2^)	Critical value	Chi (*ρ*) calculated *P* value	Inference
	Male	Female	Male	Female					
Rugae patterns									
Wavy	119	117	161	188	15	36.74	24.996	<0.001	**Significant association** ∗
Curvy	151	99	87	82					
Circular	12	14	19	21					
Straight	33	34	19	15					
Converging	7	7	10	5					
Diverging	13	18	16	3					

The asterisks were used to demonstrate values with significant difference.

**Table 5:** Association between the individual pattern (based on sides) and the tribe with respect to sex.

	Sides	Ikwerre		Igbo		DF	Chi-square (*χ* ^2^)	Critical value	Chi (*ρ*) calculated *P* value	Inference
		Male	Female	Male	Female					
Wavy	Left	61	57	79	103	3	1.55	7.81	0.67	No significant association
	Right	58	60	82	85					

Curvy	Left	78	52	49	44	3	0.51	7.81	0.92	No significant association
	Right	73	47	38	38					

Circular	Left	4	6	7	7	3	0.39	7.81	0.94	No significant association
	Right	8	8	12	14					

Straight	Left	17	20	13	8	3	1.54	7.81	0.67	No significant association
	Right	16	14	6	7					

Converging	Left	5	3	5	1	3	3.20	7.81	0.67	No significant association
	Right	2	4	5	4					

Diverging	Left	6	14	5	1	3	0.39	7.81	0.94	No significant association
	Right	7	4	11	2					

**Table 6:** Association between the tribes with the total pattern distribution (based on sides).

	Ikwerre		Igbo		DF	Chi-square (*χ* ^2^)	Critical value	Chi (*ρ*) calculated *P* value	Inference
	Male	Female	Male	Female					
Total patterns									
Left	171	152	158	164	3	0.32	7.81	0.96	No significant association
Right	164	137	154	150					

**Table 7:** *Z*-test for difference in mean length of the primary rugae of the two ethnic groups.

Variable	Sample size (*N*)	Min	Max	Mean	SD	*z* (calculated value)	|*z*| (critical value)	*P* value (calculated)	Inference
Male Igbo >5 MM	273	5.01	18.48	10.18	2.99	2.535	1.96	0.011	**Significant** ∗
Male Ikwerre >5 MM	312	5.01	19.12	9.54	3.11				

Female Igbo >5 MM	264	5.01	18.5	10.03	3.18	1.406	1.96	0.16	Not significant
Female Ikwerre >5 MM	285	5.01	17.21	9.66	2.96				

The asterisks were used to demonstrate values with significant difference.
